# Implementation of new technologies designed to improve cervical cancer screening and completion of care in low-resource settings: a case study from the Proyecto Precancer

**DOI:** 10.1186/s43058-024-00566-z

**Published:** 2024-04-05

**Authors:** Sarah D. Gilman, Patti E. Gravitt, Valerie A. Paz-Soldán, on behalf of the Proyecto Precancer Study Group, Joanna Brown, Lita Carrillo, Jhonny Cordova, Daniel Lenin del Cuadro Hidalgo, Dora Magaly Figueredo Escudero, Karina Gonzales Diaz, José Jerónimo, Alcedo Jorges, Magdalena Jurczuk, Margaret Kosek, Gabriela Ladrón de Guevarra, Renso Lopez, Andrea Matos, Diana Maria Mattos Yap, Jaime Marín, Graciela Meza, Jessica Mori, Rachel Morse, Victor Palacios Cabrejos, Reyles Ríos, Jennifer Ríos, Gessy Salva, Patricia Raquel Rivas Saurin, Karina Román, Anne F. Rositch, Hermánn Silva, Anna Smith, Carlos Santos Ortiz, Sandra Soto, Nolberto Tangoa, J. Kathleen Tracy, Javier Vásquez, Gladys Giannina Vásquez del Águila

**Affiliations:** 1 Department of Clinical Research and Leadership, The George Washington University, Washington, DC USA; 2 Department of Epidemiology and Public Health, University of Maryland School of Medicine, Baltimore, MD USA; 3 Department of Tropical Medicine and Infectious Disease, Tulane School of Public Health and Tropical Medicine, New Orleans, LA USA; 4 Behavioral Sciences Research Unit, Asociación Benéfica Prisma, Lima, Peru

**Keywords:** Implementation science, Cervical cancer, Systems-thinking, Participatory action research, Perú

## Abstract

**Background:**

This case study details the experience of the Proyecto Precancer in applying the Integrative Systems Praxis for Implementation Research (INSPIRE) methodology to guide the co-development, planning, implementation, adoption, and sustainment of new technologies and screening practices in a cervical cancer screening and management (CCSM) program in the Peruvian Amazon. We briefly describe the theoretical grounding of the INSPIRE framework, the phases of the INSPIRE process, the activities within each phase, and the RE-AIM outcomes used to evaluate program outcomes.

**Methods:**

Proyecto Precancer iteratively engaged over 90 stakeholders in the Micro Red Iquitos Sur (MRIS) health network in the Amazonian region of Loreto, Perú, through the INSPIRE phases. INSPIRE is an integrative research methodology grounded in systems thinking, participatory action research, and implementation science frameworks such as the Consolidated Framework for Implementation Research. An interrupted time-series design with a mixed-methods RE-AIM (Reach, Effectiveness, Adoption, Implementation, and Maintenance) evaluation framework was used to examine the adoption of human papillomavirus (HPV) testing (including self-sampling), with direct treatment after visual inspection with portable thermal ablation, at the primary level.

**Results:**

This approach, blending participatory action research, implementation science, and systems-thinking, led to rapid adoption and successful implementation of the new cervical cancer screening and management program within 6 months, using an HPV-based screen-and-treat strategy across 17 health facilities in one of the largest public health networks of the Peruvian Amazon. Monitoring and evaluation data revealed that, within 6 months, the MRIS had surpassed their monthly screening goals, tripling their original screening rate, with approximately 70% of HPV-positive women reaching a completion of care endpoint, compared with around 30% prior to the new CCSM strategy.

**Conclusions:**

Proyecto Precancer facilitated the adoption and sustainment of HPV testing with subsequent treatment of HPV-positive women (after visual inspection) using portable thermal ablation at the primary level. This was accompanied by the de-implementation of existing visual inspection-based screening strategies and colposcopy for routine precancer triage at the hospital level. This case study highlights how implementation science approaches were used to guide the sustained adoption of a new screen-and-treat strategy in the Peruvian Amazon, while facilitating de-implementation of older screening practices.

**Supplementary Information:**

The online version contains supplementary material available at 10.1186/s43058-024-00566-z.

Contributions to the literature

- Evidence-based recommendations for cervical cancer prevention and control in low-resource settings are established, but barriers persist around feasibility, acceptability, adoption, and sustainability given difficulties in modifying existing health systems and pre-existing CCSM practices.
- This case study demonstrates how implementation science, systems thinking, and participatory action research were leveraged to gain acceptance, adoption, and sustained use of an evidence-based strategy for cervical cancer prevention and control.
- Proyecto Precancer’s experience highlights the importance of stakeholder collaboration and program champions, the use of implementation science theories, and how academic-public health system partnerships can facilitate stronger planning, bridging implementation research and practice.

## Background

Cervical cancer is a preventable disease that remains the 4th most common cause of female cancer worldwide, with 80% of the disease burden occurring in low-and-middle-income countries (LMICs) and higher disease burdens in low-resource settings [1]. This reflects strong disparities in terms of access to care and reflects the need for adoption of effective, context-adapted prevention strategies. To address this challenge, in 2020, the World Health Organization launched a global strategy to accelerate the elimination of cervical cancer as a public health problem [2].

**Table:** 

WHO goals to be met by 2030 for countries to be on the path to cervical cancer elimination:
• 90% of girls fully vaccinated with the HPV vaccine by 15 years of age
• 70% of women screened with high-performance tests by 35 years of age, and again by 45 years of age
• 90% of women identified with cervical disease treated

With less than a decade remaining before 2030, the WHO’s proposed elimination goals require rapid scale-up of effective, sustainable, and context-adapted cervical cancer screening and management programs (CCSM) [3]. However, in practice, sustainable implementation and adoption of these programs has been elusive, reflecting challenges inherent in implementing multilevel interventions in complex adaptive health systems subject to frequent change and competing incentives [4]. This mirrors broader global challenges for health programs including non-adoption, abandonment, failure to scale, or failure to integrate sustainably into practice; challenges that occur at multiple system levels [5, 6]. Implementation science (IS) theories, models, and frameworks (TMFs), and stakeholder-engaged research approaches like systems-thinking (ST) and participatory action research (PAR) may serve to ameliorate these challenges, by increasing acceptance, adoption, and sustainability of evidence-based, locally adapted screening methods. At the same time, they may help facilitate de-implementation of technologies and systems that prove burdensome and ineffective in low-resource settings.

In Perú, cervical cancer is the second leading cause of cancer death among women [7]. Mortality rates are especially high in the country’s rainforest region, where women are less likely to have had a Pap test than women living on the coast [8] and where access to hospitals (for treatment) may depend on hours or days of boat travel on rivers. Since 1998, cervical cancer control has been a national priority [9]; however, longstanding structural barriers to Pap-based cervical cancer screening and treatment have existed throughout Perú for decades (e.g., screening capacity, sample quality and timeliness, and centralization of resources from equipment to specialists) pointing to the need for system-wide improvement that would reliably increase women’s access to screening [10].

The aim of Proyecto Precancer is to facilitate the adoption of new technologies and systems for cervical cancer screening and completion of care in the Peruvian Amazon with high cervical cancer incidence rates [6]. Proyecto Precancer (PP) is a collaboration of Peruvian and US researchers, and over 90 stakeholders from all levels of the health system in Lima (Peru’s capital), Loreto (a large region in the northeastern Amazon rainforest, Peru), and the Micro Red Iquitos Sur (MRIS) health network (the largest health network in Iquitos—Loreto’s capital) [6]. The aim of PP is to facilitate the improvement of CCSM through the adoption of new systems and technologies for cervical cancer screening and completion of care in the Peruvian Amazon.

The complexity inherent in implementing changes to the CCSM approach required engagement of multi-level stakeholders, understanding of their current system, and co-development of a guiding map for both implementers and partners. These needs, in the context of the current case study, led to the development of the INSPIRE (Integrative Systems Praxis for Implementation Research) methodology, which combined ST and PAR-based approaches with existing IS models and frameworks [3]. As INSPIRE was developed, PP iteratively sought to explore the question: “How can the use of IS models and frameworks, combined with ST and PAR approaches, influence the adoption and sustainability of new evidence-based CCSM services in a complex health system located in the Peruvian Amazon?”.

**Fig. 1:**
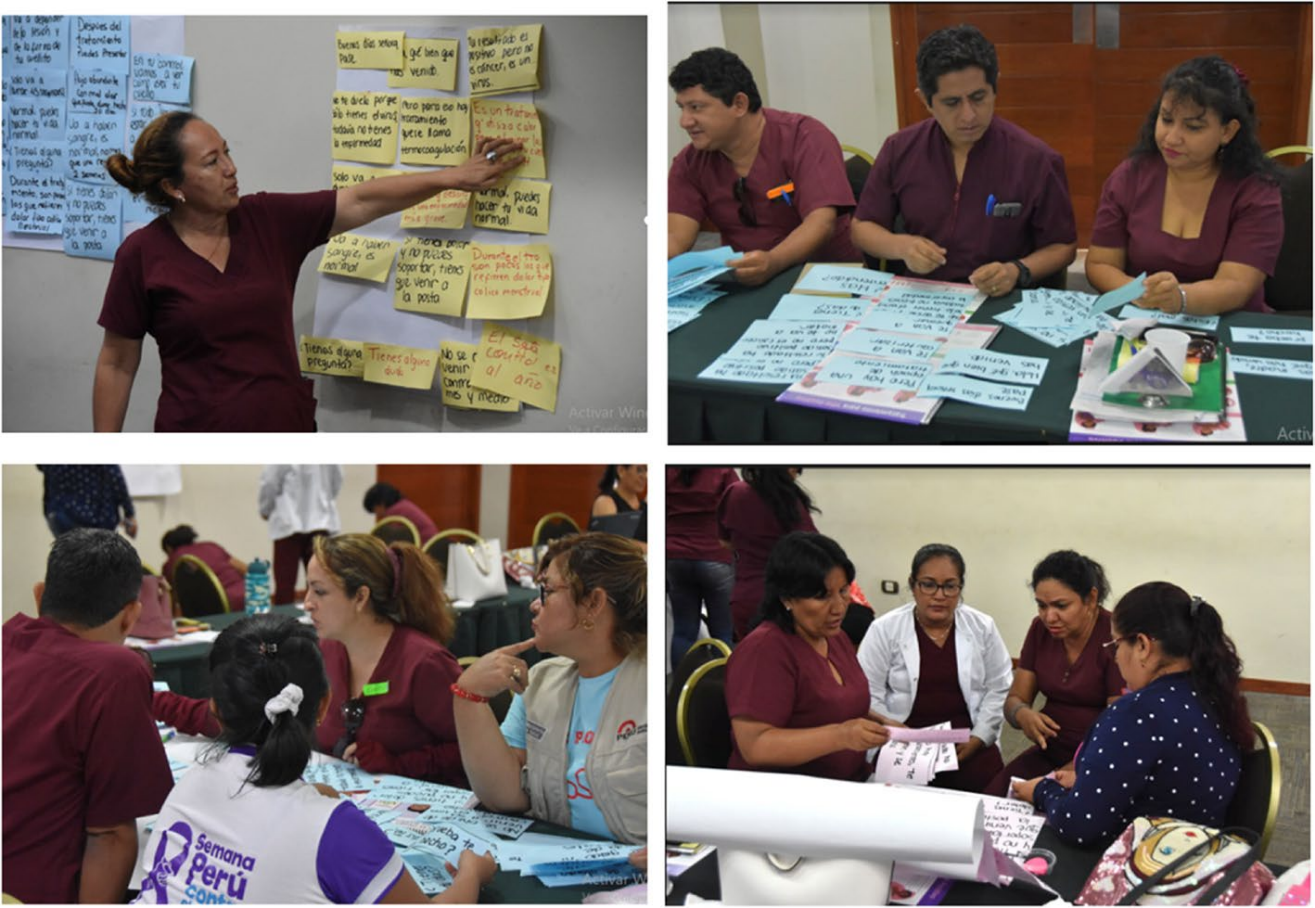
PP partners mapping their CCSM system

The aim of this case study is to detail PP’s use of a participatory, ST and IS-guided process with stakeholders throughout the MRIS health system—and health authorities at regional and national levels—to guide the implementation, adoption, and sustainment of new technologies for CCSM in the Peruvian Amazon (see Fig. 1).

## Methods

### Case study setting

The MRIS network covers a population of 150,000, with a target population of 20,000 women 30–49 years of age eligible for cervical cancer screening via HPV testing. Comprised of urban, peri-urban, and rural communities, Iquitos (~ population 400 K) is the largest city in the world that is accessible only by air or river. Within Iquitos, transportation routes include networks of paved and unpaved roads that connect communities to each other, one main paved highway, and boat travel by river.

### Approaches and frameworks that informed INSPIRE

INSPIRE arose from a constellation of needs that included visualizing the health system from multiple perspectives, engaging stakeholders at all system levels, identifying barriers and facilitators to change, determining how to adapt knowledge to the local context, and planning implementation while de-implementing existing but less effective practices. Based on these needs, PP combined three approaches—IS, PAR, and ST—to maximize opportunities for effective translation of evidence to practice in CCSM in this context [3]. PP leveraged IS theories, models, and frameworks (TMFs) to: (1) guide translation of HPV-based screen and treat methods from research to practice, (2) understand influences on HPV-based screen and treat outcomes, and (3) monitor and evaluate the implementation of the screen and treat program [11] (see Tables 1 and 2, and Additional file 1, for detailed descriptions of TMF and approaches used in different INSPIRE phases). The Consolidated Framework for Implementation Research (CFIR) [3, 6] was selected as a determinant framework to guide researchers in the process of knowledge creation (i.e., multiple and varied constructs on determinants of implementation to observe and document) and application, facilitating proper fit to context in the adaptation process. All CFIR constructs were not used, but as the PP team tried to document a dynamic and complex multilevel system change, CFIR was helpful for sensemaking and organizing data. The RE-AIM (reach, effectiveness, adoption, implementation, maintenance) framework [12–14] was selected to guide evaluation, by defining key indicators and issues to monitor over time—both quantitatively and qualitatively. While the CFIR and RE-AIM frameworks fit best in this particular context, they were not the only determinant or evaluation frameworks that could have served, and research teams in other settings may consider alternate frameworks, so long as they plan to include a determinant and evaluation framework to help operationalize INSPIRE.

**Table 1:** Approaches, models, and frameworks that help explain the mechanisms behind INSPIRE

Name	Type	Purpose
*Consolidated Framework for Implementation Research (CFIR)*	Determinant	To classify barriers and facilitators to implementation in a multi-level, complex health system that surfaced during the INSPIRE methodology, allowing appropriate matching of implementation strategies to context and more generalizable reporting of results
*RE-AIM (reach, effectiveness, adoption, implementation, maintenance) Framework*	Evaluation	To assess the potential influences that INSPIRE had on CCSM practices throughout the MRIS health network
*Systems thinking*	Theoretical approach	To converge stakeholder mental models of complex health care systems involving multiple perspectives to facilitate shared decision making, action, reflection, and adaptation
*Participatory Action Research*	Action research approach	To encourage stakeholder engagement, co-learning, and co-production of knowledge

*scaling up: realist evaluation of HPV screen and treat strategy in Amazon basin of Peru*

**Table 2:** Integration of research methods, implementation science (IS) frameworks, and implementation strategies by INSPIRE phase

	**INSPIRE ACTION**	**RESEARCH METHODS UTILIZED**	**IS FRAMEWORKS**	**IMPLEMENTATION STRATEGIES**
INSPIRE HUB	1. Define problem situation with stakeholders	Soft systems methodology	*CFIR Domain 1*: intervention source	Build buy-in (involve existing governance structures, ID champions) Develop relationships (build coalitions, resource-sharing agreements, formal commitments, academic partnerships)
			*CFIR Domain 5:* engaging	
	2. Launch the project			
PHASE 1: UNDERSTAND THE SYSTEM	3. Developmental models of the system	AIIM SAST stage 1 with key informant interviews and focus group discussions KAP surveys Audits of current system outcomes Pathway analysis visually represented by flow charts and swim-lane diagrams	*CFIR Domain 3*: defining structural characteristics, networks, & communications	Gather information (needs assessment, readiness to change)
				Involve patient/consumers and family members
	4. Establish narrative and stakeholder perceptions of the system			
			*CFIR Domain 2:* culture & implementation climate	
				Audit current system behavior Capture and share local knowledge
			*CFIR Domain 3:* patient needs and resources *CFIR Domain 3:* external policies & incentives	
			*CFIR Domain 4:* knowledge & beliefs about the intervention	
			*CFIR Domain 4:* understand self-efficacy, individual stage of change and other attributes	
	5. Make the system visible			
PHASE 2: FIND LEVERAGE	6. Engage stakeholders in group model building	SAST Stage 2 – design workshops Dialectic debate and group model building (facilitated with goal to balance desirability and feasibility guided by reflection on implementation outcomes such as feasibility, cost, acceptability, sustainability, etc.) Scenario analysis	*CFIR Domain 1:* review characteristics of the intervention and options (evidence strength and quality, relative advantage, complexity, cost) & assess adaptability and trialability of alternatives *CFIR Domain 3:* assess cosmopolitanism, peer pressure, influence of external policies/incentives *CFIR Domain 4:* assess KAB about intervention options *CFIR Domain 4:* Group level stage of change	Assess readiness and identify barriers Get feedback from audit of current system behavior Purposefully re-examine the intervention Tailor strategies to overcome barriers and honor preferences Model and simulate change Conduct local consensus discussions Distribute educational materials and conduct educational meetings Make training/education dynamic and participatory Inform local opinion leaders Create a learning collaborative Consider restructuring strategies as leverage opportunities Consider financing strategies as leverage opportunities Mandate change
	7. Share, test, revise system/process maps			
	8. Define and localize system behaviors contributing to problem situation			
	9. Find leverage for change			
PHASE 3: ACT STRATEGICALLY	10. Stakeholder designed implementation plan 11. Infrastructure modifications, training, dissemination plan development 12. Implement changes	Work group soft systems methodology with research team facilitation	*CFIR Domain 1:* design quality *CFIR Domain 1:* complexity *CFIR Domain 5:* planning *CFIR Domain 5:* executing	Develop a formal implementation blueprint Tailor strategies to overcome barriers and honor preferences Stage implementation scale-up Involve patients/consumers and family members Recruit, designate, and train for leadership Obtain formal commitments Develop effective educational materials relevant to mandated change Develop a glossary of implementation (including new models) Distribute educational materials Conduct ongoing, dynamic training Conduct educational outreach visits Use train-the-trainer strategies Provide ongoing consultation Place new interventions on fee for service lists/formularies *Develop supply chain management* Revise professional roles Create new clinical teams Change services sites Change equipment Change records systems Develop and organize quality monitoring systems Develop tools for quality monitoring Use advisory boards and work groups Conduct cyclical tests of change Create or change credentialing and/or licensure standards
PHASE 4: LEARN AND ADAPT	13. Ongoing M&E using stakeholder defined implementation outcome metrics 14. Share M&E with stakeholder group 15. Re-initiate INSPIRE cycle where indicated by identification and localization of new or unresolved problem situation	M&E for primary implementation outcomes SAST with KII and FGD Design workshops	*RE-AIM* *CFIR Domain 5*: Reflecting and evaluating	Provide ongoing consultation Sustain a learning collaborative Use mass media to increase reach *(only after system behavior is stabilized post-implementation)* Use advisory boards and working groups Organize clinical implementation team meetings

Reproduced from *Cancer Epidemiology, Biomarkers & Prevention.* 2020. Vol.29,9, 1710–1719, Gravitt, P. et al., “Integrative Systems Praxis for Implementation Research (INSPIRE): An Implementation Methodology to Facilitate the Global Elimination of Cervical Cancer., with permission from the American Association for Cancer Research (AACR)

Abbreviations: *AIIM*, alignment, influence, and interest matrix; *CFIR*, Consolidated Framework for Implementation Research; *DW*, design workshop; *FGD*, focus group discussions; *HSF*, health systems framework; *ID*: Infectious Disease; *KAB*, knowledge attitudes and beliefs; *KAP*, knowledge, attitudes, and practices; *KII*, key informant interview; *SAST*, strategic assumption surfacing and testing

PAR and ST approaches were also central to the way the research team engaged with PP champions and stakeholders, and the dynamic health system. Establishing trust and mutual respect with stakeholders was critical to enhance the chances of making context-relevant decisions, ultimately promoting adoption and sustainability. PAR approaches hinge strongly on stakeholder engagement, co-learning, and co-production of knowledge and potential solutions [15]. Hence, the research team ensured PAR was central to all interactions—from understanding system barriers to decision-making. ST can be particularly helpful for developing clear mental models of dynamic and complex systems (such as health care systems) that involve multiple perspectives, a range of systems levels, and interacting agents that evolve over time [16, 17]. ST served as a powerful tool to understand multidimensional barriers and facilitators to CCSM in this setting, by ensuring that as stakeholders made changes in one part of the health system, they also anticipated and planned for other resulting changes in the system.

### The INSPIRE Model

Bringing these approaches and frameworks together, INSPIRE consists of four key phases:

- Phase 1: Understand the system
- Phase 2: Find leverage
- Phase 3: Act strategically
- Phase 4: Learn and adapt

**Fig. 2:**
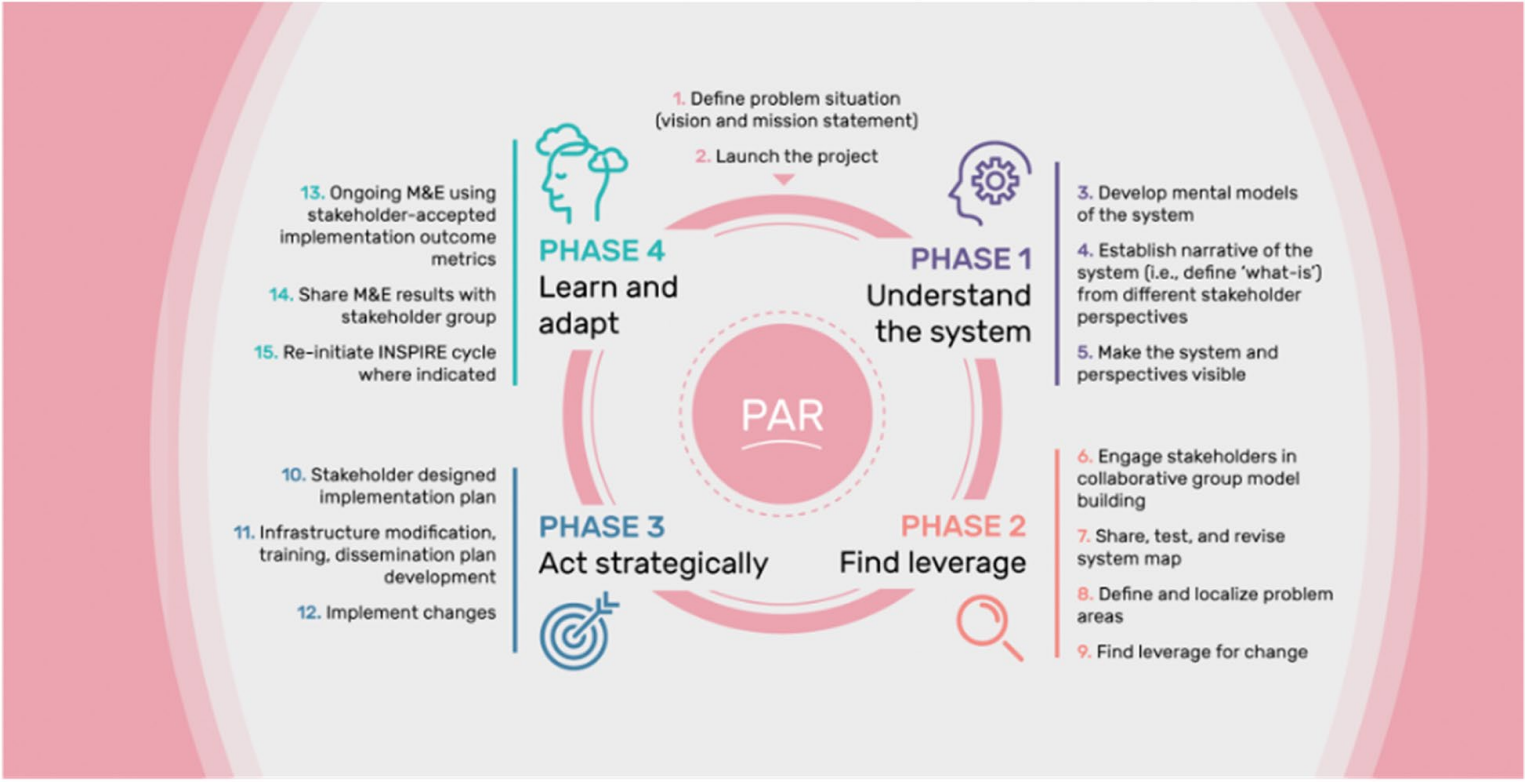
The INSPIRE Model

Each phase of INSPIRE (see Fig. 2) relies on the use of these ST, PAR, and IS frameworks, along with defined implementation strategies to operationalize each of the phases and meet its objectives.

### Case study methods by INSPIRE phase

**Fig. 3:**
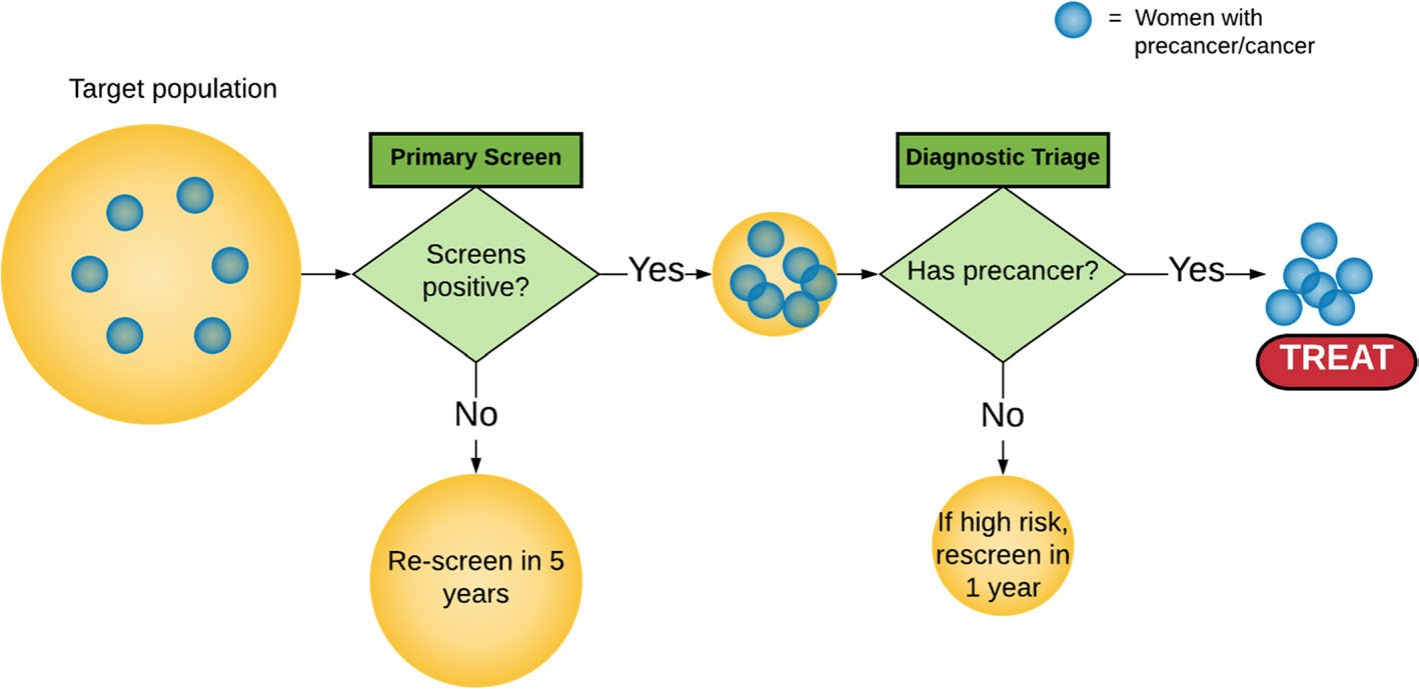
Mental model of an idealized CCSM system

**Fig. 4:**
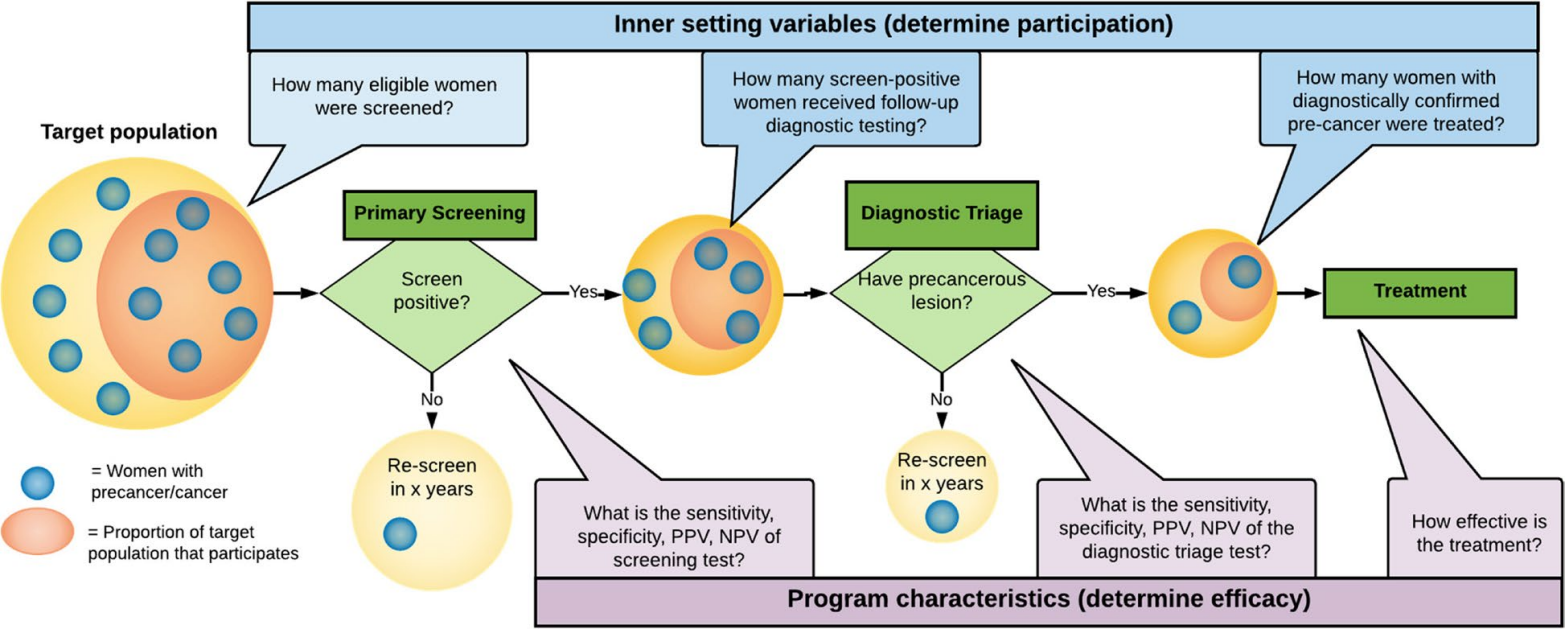
Mental model of a more realistic CCSM system with key considerations

In Phase 1, interviews and focus groups with different system stakeholders were conducted to generate a detailed visual of the system and all its complexity from multiple perspectives. Through system audits and process mapping the CCSM system in a visual manner, PP moved stakeholders from “solving” or “judging” parts of the system to “understanding” that all were part of a complex system with multiple barriers: fragmentation, duplication, delays, insufficient training among health professionals on screening and triage techniques, and lack of a monitoring system. This process enabled a shared understanding of current (see Fig. 3) and ideal systems (see Fig. 4) to emerge, and also led stakeholders to comprehend that all parts of the CCSM mental model were necessary for the system to work effectively: for example, by acknowledging that screening without clinical follow-up was useless. During this phase, program champions emerged as key stakeholders who were interested and engaged in all processes and that were critical in solving local challenges.

**Fig. 5:**
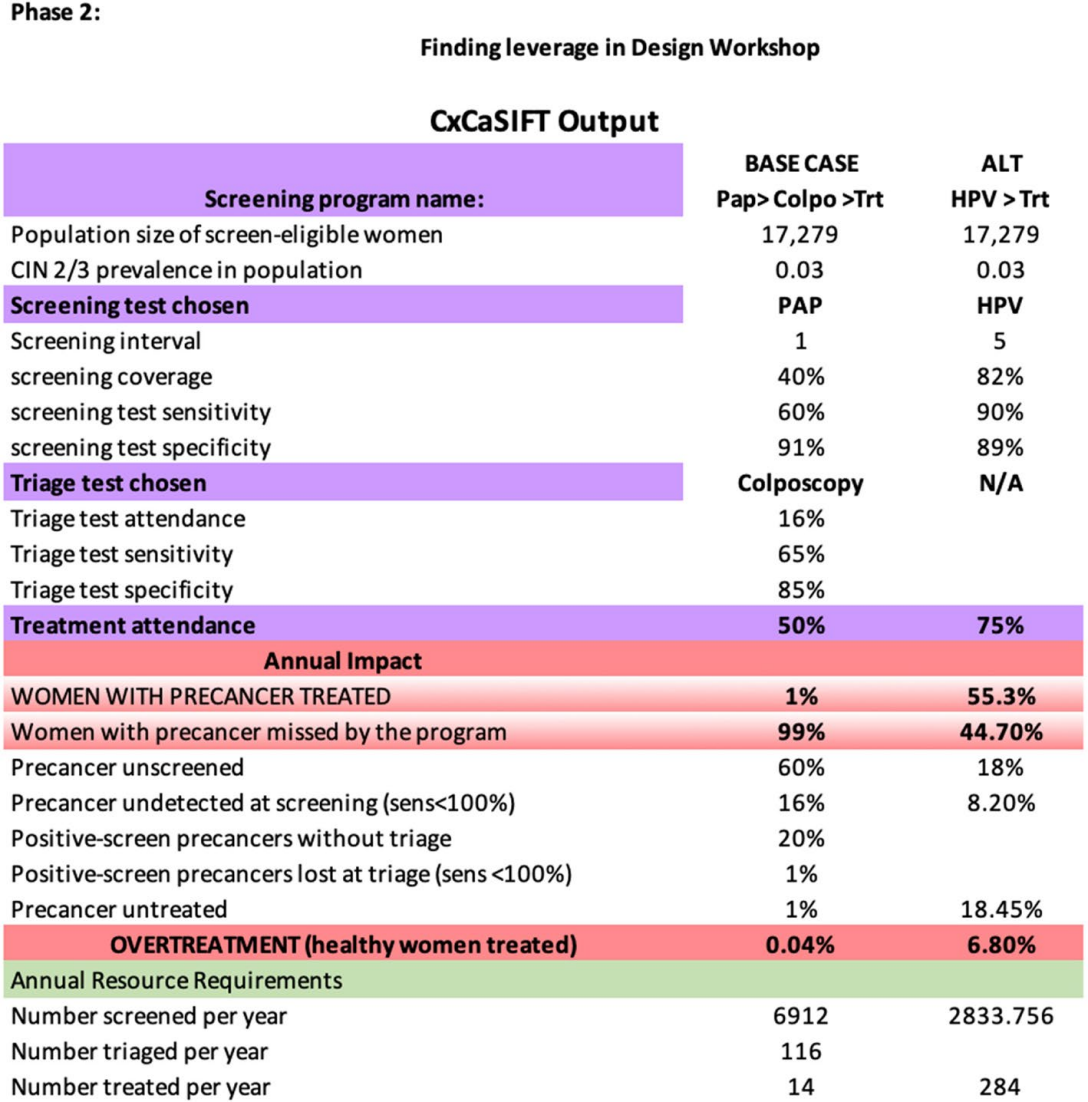
Example of scenario analysis tool output during phase 2

In Phase 2, PP engaged multilevel stakeholders in group model building (GMB) workshops [18]: sharing, testing, and revising system maps, and then defining and localizing problem areas, including bottlenecks, fragmentation, and duplication. The GMB—focused on a shared vision of what *could* happen in Iquitos—enabled the group to ultimately find leverage for change. In these GMB workshops, PP chose to focus specifically on a combination of deliberative dialogue [19] and scenario analysis [20–23] to test assumptions in real time, using a collaborative, exploration-focused approach that helped to bring about a shared vision for future implementation. In scenario analysis, stakeholders and researchers examined simple models of several evidence-based CCSM approaches. Specifically, based on assumptions that could be modified in real time, stakeholders could observe the predicted trade-offs of various approaches (i.e., observing how one screening or triage decision could affect the total number of women with precancer or cancer who are ultimately identified or treated), helping them define a preferred CCSM approach for adoption and evaluation (see Fig. 5 for an example of scenario analysis tool output).

During GMB and based on the setting realities, PP stakeholders felt that HPV-based screening (including patient self-collection) could reduce barriers presented by cytology-based screening programs, with the added benefit of highly sensitive and objective results, and lower monthly screening goals (e.g., screening every 5 years instead of 3 years). Additionally, referring all positive women to the regional hospital with limited availability of colposcopy triage appointments created significant bottlenecks for continuum of care. Hence, system stakeholders agreed to manage all eligible HPV-positive women at primary health facilities, with a nurse midwife providing counseling and education, and a trained medical doctor conducting visual triage for treatment and managing all eligible women with ablative therapy. Medical doctors at the primary level were trained and observed (approximately 10–15 times) by gynecologists with expertise in ablative therapy. Thermal ablation was deemed more feasible than cryotherapy due to ease of transport and charging. Only women with suspected cancer, large lesions (> 75% of transformation zone), or a transformation zone that was hard to see were referred to hospital specialists. Stakeholders ultimately decided on this task-shifting approach for adoption in Iquitos and were confident about this decision due to the shared knowledge about evidence and options available to them.

In Phase 3, PP focused on strategic action, developing stakeholder-designed implementation plans (via stakeholder working groups), and conceptualizing infrastructure modifications. This included adapting an internal hybrid monitoring and evaluation (M&E) system to evaluate impact, working with authorities to add new services to the public health billing system, adapting laboratory space for sample processing, and helping the health system to prepare for task-shifting and training health care providers (including lab technicians, midwives, medical doctors at the primary level, and specialists). Additional strategic actions included developing dissemination plans and planning key program adaptations. Across all phases, but especially in phase 3, PP bundled discrete implementation strategies to meet stakeholder needs across the implementation continuum (acceptability, adoption, implementation, scale-up, and maintenance). For example, three working groups independently met to plan for changes at the primary screening level (i.e., HPV screening and counseling needs); for thermal ablation, triage, and treatment (i.e., system flow, training needs); and for hospital-level referrals and management. Each working group produced a plan describing, not just what activities should be completed at every phase, but by whom and how they should be completed.

In Phase 4, the focus was on deliberative dialogue to establish ongoing M&E using stakeholder-accepted outcomes, followed by review of M&E results with stakeholders, and re-initiation of the INSPIRE cycle where indicated. For example, when screening increased significantly in urban health facilities but not rural health facilities, key stakeholders met to discuss a range of challenges and potential solutions (phase 1), find leverage in the system (phase 2), and to plan and implement possible solutions and make adaptations (phase 3). In this example, community campaigns in rural areas with self-collection were undertaken to reach women in their homes.

### Intervention, adaptation, and implementation strategies

From March 2017 to June 2019, the project focused on INSPIRE phases 1–3. This included interviews and system audits, group model-building workshops with stakeholders throughout the health system, establishing a patient-level screening registry, implementation planning workshops, and preparation for implementation of the CCSM program among women ages 30–49, eligible for HPV-based testing (see Supp file, Table 2). Phased implementation of the new CCSM strategy implementing HPV testing and thermoablative treatment at the primary level (concurrent to de-implementing visual inspection with acetic acid (VIA) and Pap with referral to hospital level follow-up with colposcopy)—took place from July 2019 to the present, albeit with interruptions due to COVID-19 and supply-chain issues with testing supplies. Evaluation of the program (Phase 4 or “learn and adapt”) is ongoing to contribute to the iterative improvement of the program.

Program adaptation to ensure fit with the local context was critical to adoption. For example, prior to PP, health professionals filled out numerous paper-based forms for billing, clinical records, and patient follow-up. The PP team facilitated the design of a triple-copy form that could meet the multiple documentation needs of the health system, and that could also be used for the hybrid electronic-paper patient-level screening registry to ensure adequate tracking of cases and reduction of loss to follow-up. The de-implementation of Pap-smear and VIA as a screening method, and the introduction of HPV testing that included patient self-collection, also required new nurse-midwife trainings and health education materials, and new approaches to health counseling. Additional adaptations included modifying Ministry of Health billing practices to allow for new procurement processes related to CCSM purchases and supplies, changes to the insurance reimbursement process, and adaptations to the screening processes in rural areas, using campaign-style methods.

### Measurement and design

**Fig. 6:**
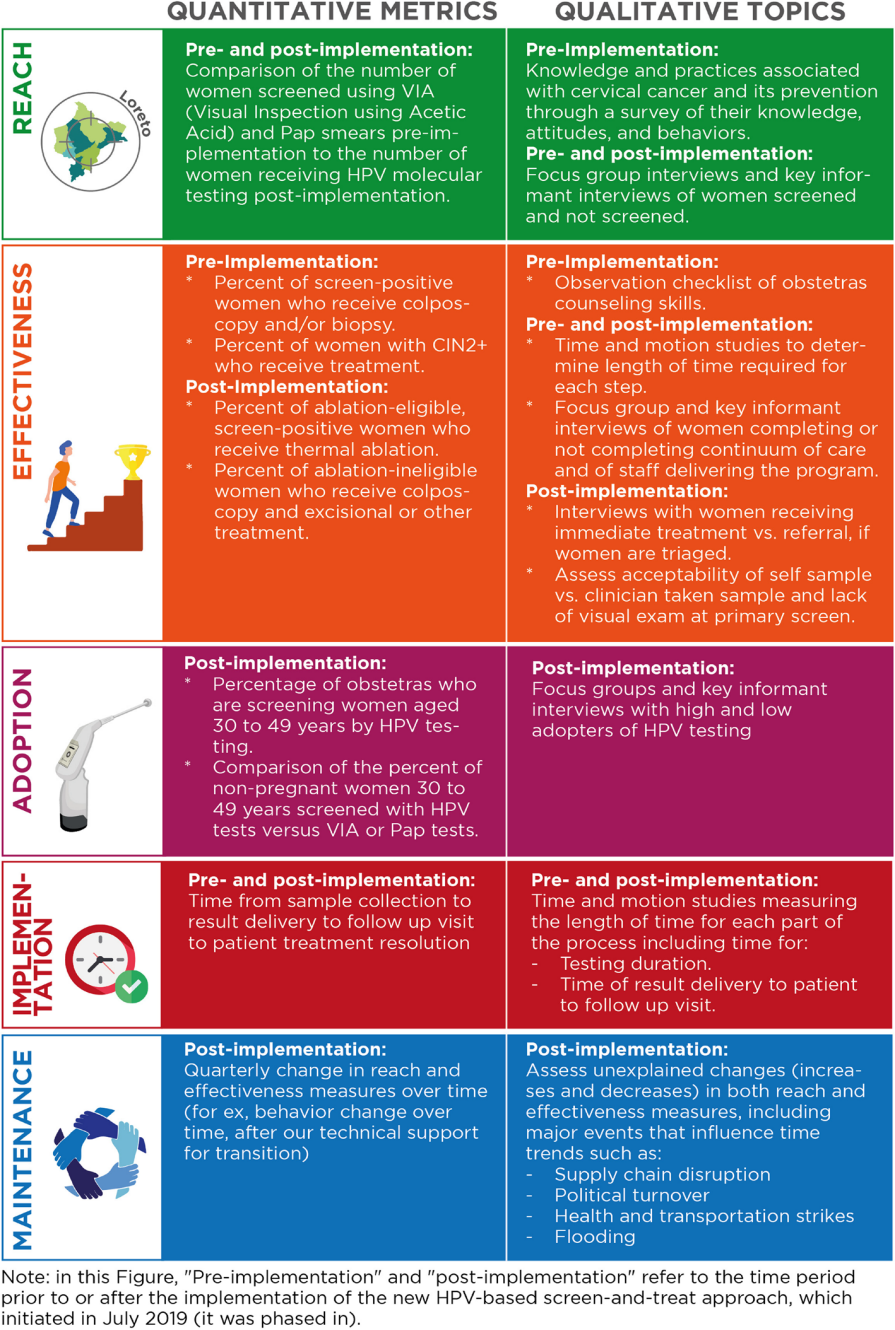
Quantitative metrics and qualitative topics examined both pre- and post-implementation, using the RE-AIM model

This implementation research study used an interrupted time series design with a mixed-methods RE-AIM evaluation framework (see Fig. 6). Qualitative research was used throughout the project (see Table 3): initially to understand the system challenges in depth, but as new issues emerged (for example, when screening in rural areas did not increase relative to urban areas), these triggered new rounds of interviews and focus groups that allowed us to work with stakeholders to understand the relevant issues (e.g., acceptability, adoption), and reflect and respond appropriately. As new challenges emerged, we responded by initiating short cycles of INSPIRE phases 1 through 3 to understand the problem, determine ways to address it, and plan and implement it. Having all but one of our research team members based in Peru facilitated this continuous monitoring and communication.

**Table 3:**
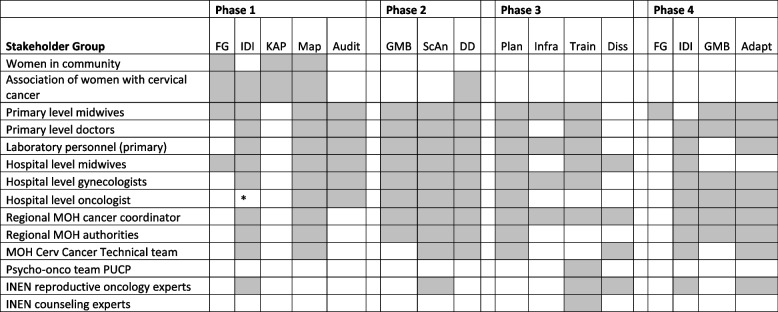
Stakeholder involvement by phase and activity

Abbreviations: *FG* Focus groups, *IDI* in-depth interviews, *KAP* Knowledge, Attitudes and Practices, *Map* mapping, *MOH* Ministry of Health, *GMB* Group model building, *ScAn* Scenario analysis, *DD* Deliberative dialogue, *Diss* Dissemination plan development, *Adapt* Adaptation. *Indicates no oncologist available

### Reach

Reach was assessed quantitatively, by estimating coverage targets as monthly unique screening tests in women aged 30–49 years old, based on the WHO goal of encouraging screening for 70% of 20,000 over a 3-year (VIA) or 5-year (HPV) period (*n* = 389/month and 233/month, respectively). The percent of monthly target screening tests was tracked using VIA and compared to the percent of participants screened using HPV-based testing. Change in screening rates in the pre-implementation time period (Jan 1, 2018–Jun 30, 2019) compared with the post-implementation time period (July 1, 2019–Feb 27, 2020) was then evaluated in an interrupted time series analysis with Poisson regression, modeling screening counts and adjusting for seasonal variation. Fourier terms and a scaling parameter were included to allow for over-dispersion of data. Qualitatively, reach was assessed by mapping barriers and facilitators identified through a knowledge, attitudes, and practices (KAP) survey and through focus-group and key-informant interviews with women screened and not screened.

### Effectiveness

Effectiveness was evaluated by tracking data on the proportion of women who completed the CCSM continuum of care, comparing the proportion of Pap/VIA screen-positive women who attended colposcopy with the proportion of HPV screen-positive women receiving thermoablative treatment for management of pre-cancerous lesions, or referral to colposcopy (based on documentation that women received colposcopy) if ineligible for ablation. Additionally, semi-structured interviews were conducted with multilevel stakeholders at varying time points pre- and post-implementation (see Fig. 6) due to the fluidity and complexity of this system change: as challenges were identified, the research team collected data to understand the problem. For example, interviews were conducted with women who were lost to follow-up before and after implementation of the new approach to understand barriers in the system, as well as the potential role of stigma in generating barriers to seeking screening or care.

### Adoption

To measure adoption, PP collected data on the proportion of health facilities that utilized HPV-based testing methods to screen women ages 30–49. Proportion of total screens for VIA as compared to HPV-based testing were also compared over time. Focus groups and in-depth discussions were also held with midwives representing a range of settings within this network: urban, peri-urban, and rural. Additional topics explored in in-depth interviews and focus groups examining acceptability, appropriateness, feasibility, fidelity, penetration, and sustainability associated with changes in the system.

Regarding visual triage for treatment, per current national regulations, treatment must be conducted by medical doctors. Most health facilities in the MRIS (and in the country) do not have medical doctors, and there are insufficient funds to consider thermocoagulators in every health facility. Thus, the working group opted for setting up two MRIS health facilities with trained doctors to conduct visual triage and thermal ablative treatment: one health facility opted for offering this continuously, whereas the other set a date (every Thursday) for this procedure.

### Implementation

Implementation measures focused on time and motion studies, evaluating time from sample collection to testing, result delivery to patient, and result delivery to follow-up visit. Qualitative research explored causes of system bottlenecks (addressed by initiating INSPIRE Phase 1 for that particular issue).

### Maintenance

To evaluate potential for maintenance, PP assessed changes in reach and effectiveness measures over time and qualitatively noted any events in the broader context that might influence or disrupt time trends (for example, supply chain disruptions, COVID-19, or political turnover). This also included interviews and discussions at the national level, who were closely engaged in the processes in Loreto: the Ministry of Health provided suggestions about topics that might be challenging to implement or might get resistance from national stakeholders (for example, whether thermal ablation would be an acceptable follow-up approach for specialists from the capital city of Lima with different resources available), and the PP team would generate evidence that was needed at a national level for decision-making.

The Standards for Quality Improvement Reporting Excellence guidelines were used as a framework for reporting this case study [24]. A table mapping the guidelines to each section of the manuscript is included below in Additional file 3 (SQUIRE checklist).

## Results

### Stakeholder engagement

More than 90 stakeholders—from MRIS midwives or lab technicians to regional and national health authorities and hospital level specialists—collaborated with PP throughout INSPIRE (see Table 3 for additional details on engagement for each phase of INSPIRE).

### Reach

PP M&E data showed that almost 3000 HPV screening tests were collected among the primary intervention target group (non-pregnant women aged 30–49 years), with approximately 20% of these (602) positive for HPV [25]. Among the primary intervention target group (non-pregnant women aged 30–49 years), PP M&E data showed that screening rates surpassed 70% monthly coverage targets established according to WHO’s elimination goals within 6 months. In comparison, prior to HPV testing, only 31% of women were being routinely screened with either Pap or VIA [25, 26]. Qualitative findings for reach, measured prior to implementation, indicated that facilitators to screening-seeking behaviors included having knowledge about cervical cancer, having partner support, attending a health post, and receiving information from a health post while, among women not previously screened, fear was the primary barrier [27].

### Effectiveness

**Fig. 7:**
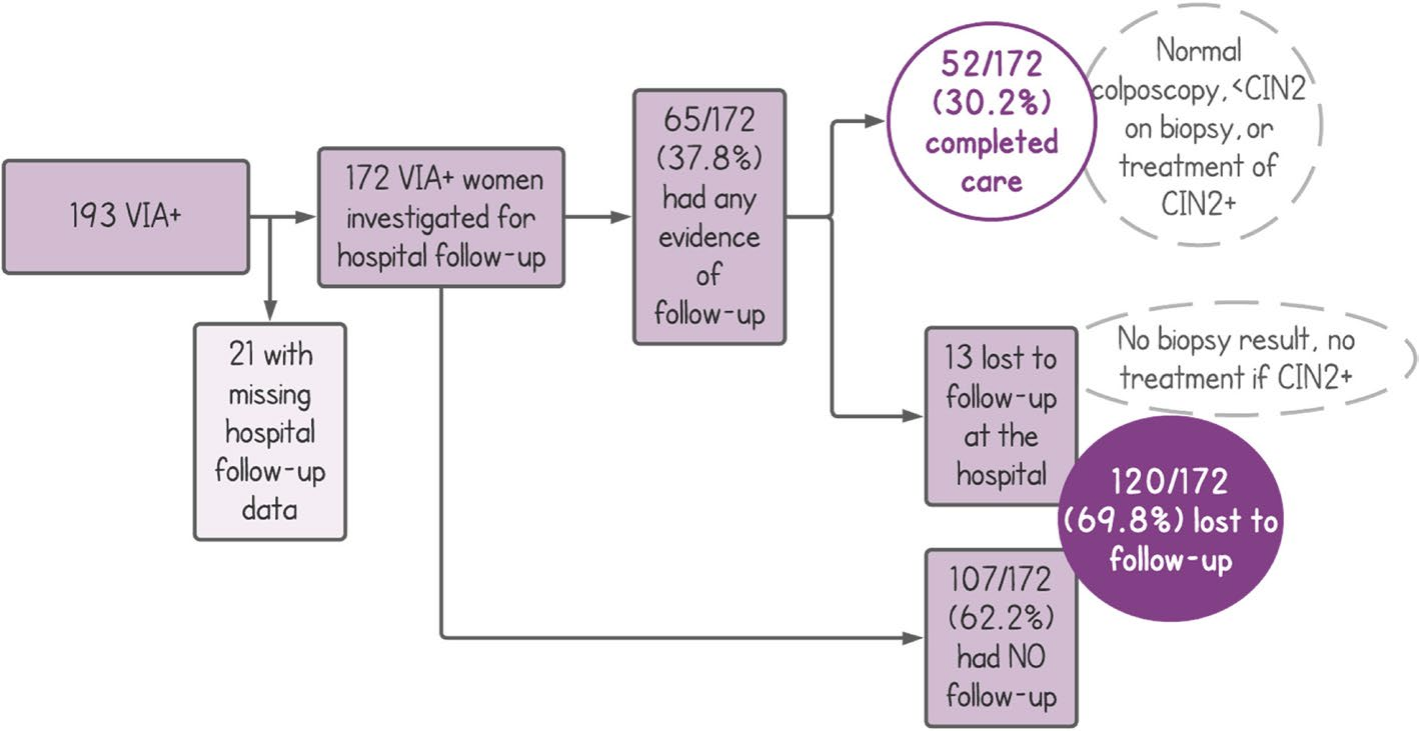
Phase 1—Understanding the system (pre-implementation)

**Fig. 8:**
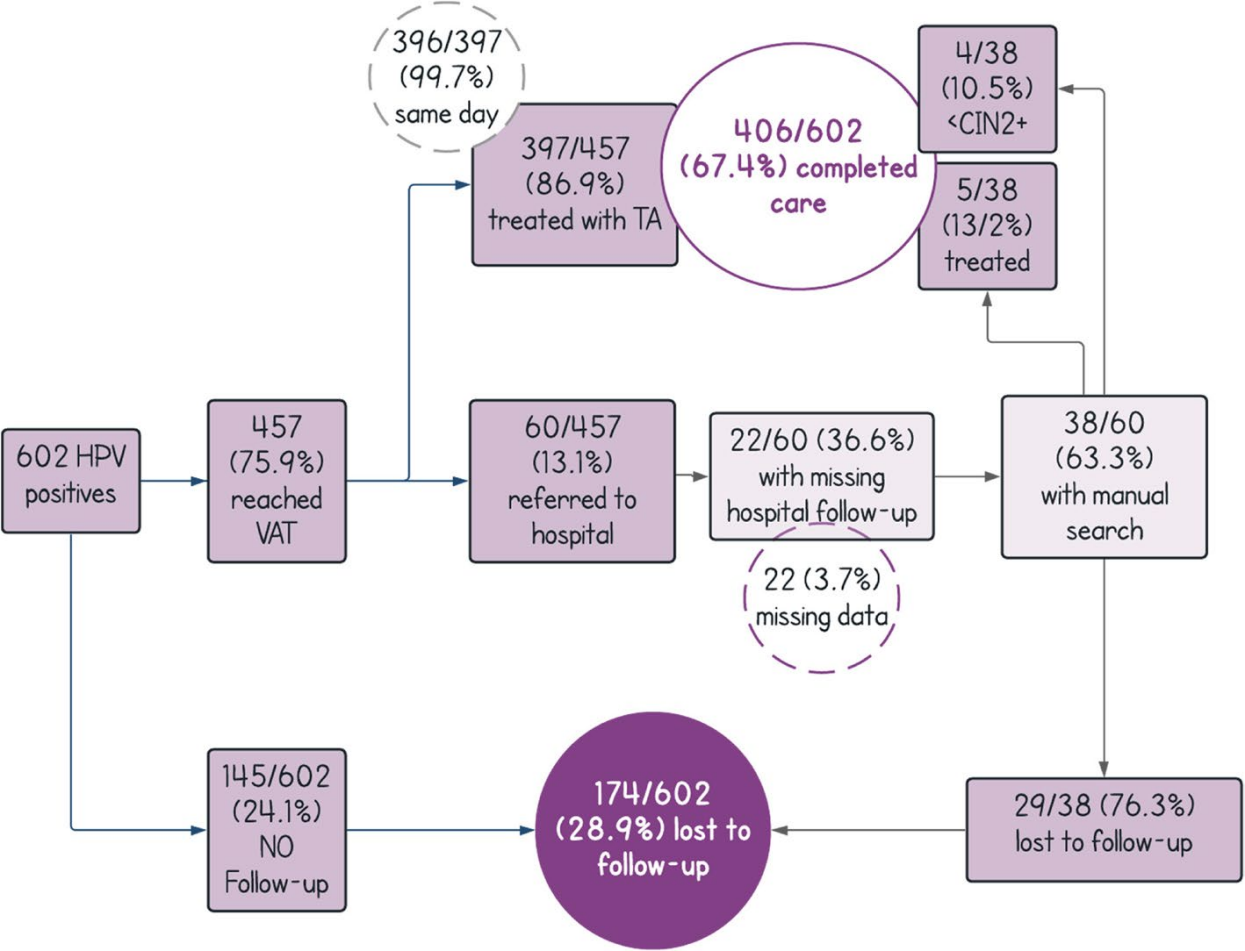
Phase 4—Learn and adapt (post-implementation)

PP M&E data show that a completion of care endpoint (interpreted as receiving ablative therapy for precancerous lesions or referral to colposcopy if ineligible for ablative therapy) was reached by 67.4% (406/602) of HPV-positive women within 6 months, compared with 30.2% (52/172) of VIA-positive women at hospital facilities (*p* < 0.001) [24]. Figure 7 (pre-implementation) and Fig. 8 (post-implementation) compare pre-and-post-implementation results for completion of care and loss to follow-up. Qualitative research conducted pre- and post-implementation (see Fig. 6) provided valuable information on the acceptability of self-collection, improvements that could be made to the counseling process and within the care continuum. It was particularly helpful to explore women’s experiences with self-collection, and before, during, and after treatment with ablative therapy. Findings indicated strong support for both modalities [28]. Women found both self-collection and ablative therapy to be accessible and acceptable: up to 90% of women in rural areas self-collected, compared to approximately 60% in urban areas. All women had the choice of visual triage to determine eligibility for thermal ablation at the primary level versus seeking a specialist referral to the hospital: all (100%) chose visual triage at the primary level. Additionally, participants who received ablative therapy stated emotional satisfaction of a swift and accessible resolution at local primary health facilities [28].

### Adoption

The HPV screen-and-treat intervention demonstrated rapid adoption within 6 months across 14/17 health facilities in the Micro Red Iquitos Sur (MRIS) health network. In the same time period (July 2019–November 2019), VIA screening was de-implemented in all 17 primary health facilities [3]. In the case of the 3/17 health facilities that did not meet adoption criteria, these health centers did not satisfy requirements for monthly HPV-based screening; however, these facilities continued to have screening rates surpassing those observed before the implementation of HPV-based testing. Post-implementation focus groups and key informant interviews yielded important information regarding high and low adopters of HPV testing, for example the impact of geographic context and health champions.

Moreover, in addition to screening, as described earlier, two health facilities served as thermal ablation centers. One health facility offered ablative therapy on a daily basis to HPV + women who were eligible and had been referred to them, and the other health facility offered a thermal ablation clinic on Thursdays. This was an example of local adaptation, with each health facility making different decisions based on its own unique needs and contexts. One of these teams later began offering travel clinics, meeting women at rural health clinics to circumvent structural barriers to screening posed by geographic and transportation challenges. PP interpreted these local and continued adaptations as evidence of health-system ownership and program success.

### Implementation

Time and motion studies conducted pre- and post-implementation (see Fig. 6) found that the median time per participant at in-clinic HPV screening visits was 3 h and 6 min (from arrival to departure) [29]. HPV test results were available within a median of 7 days (interquartile range 4–13). This can be partially explained by the fact that MRIS facilities sent samples to a shared lab at the primary level within the public health network. Samples were typically sent in bundles, as health centers often waited until they had sufficient samples to send to the lab. Median time spent at the visual assessment with ablative treatment was 2 h and 40 min (from arrival to departure) [29]. In contrast, the median time for delivery of Pap results was 20 days. HPV sample collection and ablative treatment required only a median of 5 min and approximately 20 min for registration and counseling per patient, indicating the potential to reduce wait times [29]. While the time and motion studies indicate that some improvements are needed, they also clearly demonstrate improvement in the system, and show how this type of data can be used to iteratively improve system learning, planning, and sustainment, especially when paired with simulation models and ongoing quality improvement discussions [23, 29].

Interviews and time and motion data, when combined with the quantitative data on reach and adoption, revealed that task-shifting management of screen-positive women to primary health facilities significantly improved quality of care, reduced loss to follow-up, and improved completion of care.

### Maintenance

Regional support for the program remains high and includes the organization of stakeholder-driven adaptation workshops and a regional proclamation by policymakers, prioritizing the regional reduction of cervical cancer. Through political upheaval (Perú has had seven presidents between 2016 and 2023) and one instance of national-level health authority pressure to return to the previous Pap-based screening mode (over a period of 6 months), regional authorities and health professionals in Iquitos have continually asserted that the HPV-screen-and-treat strategy is most feasible for their context.

Interviews and focus groups, conducted post-implementation, support the observation that the HPV screen-and-treat program in Iquitos, Peru, was impacted by, but ultimately resilient to, pandemic service disruptions, political instability, flooding, health system strikes, and supply chain disruptions, suggesting a high probability of sustainability over time (see Fig. 6). Iquitos was one of the world regions hardest hit by COVID-19 between March and June 2020, with a COVID-19 prevalence of 70% by July 2020, despite lockdowns due to COVID-19 [30]. The CCSM program was paralyzed for 3 to 4 months, but resumed fully by August 2020, without outside intervention. Using evidence generated by this program, the Peruvian Ministry of Health is scaling up beyond the Micro Red Iquitos Sur health network to the entire state of Loreto, and to 14 new regions of Perú located in the Amazon, on the coast and in the mountains, using this NCI-funded Iquitos experience as a national pilot.

## Discussion

Through visualization of the health system and the processes and interrelationships that influence it, and with continuous engagement with stakeholders, PP facilitated adoption of self-sampling, molecular-based primary cervical cancer screening, and direct treatment (after visual assessment) with portable thermal ablation. This includes the de-implementation of existing visual inspection-based strategies and colposcopy for routine precancer diagnosis.

This case study underscores the scientific relevance of employing IS approaches in global health, in conjunction with ST and PAR, to help us reach the WHO’s cervical cancer elimination goals. By adopting, implementing, and adapting feasible and effective screening and management strategies (while facilitating de-implementation of less feasible approaches), a more equitable health system can emerge for the women it serves [4]. The INSPIRE methodology offers a phased process that can be adapted to different topics and settings, while using PAR, ST, and IS to achieve the objectives of each phase. IS TMFs—such as CFIR and RE-AIM—were useful tools for engaging in sense-making throughout a complex and dynamic implementation experience, allowing PP to bridge implementation research and practice in a meaningful way. Since the end of this project, CFIR has been updated. These updates include the addition of determinants to equity in implementation and improved centering of innovation recipients [31].

This program offers a good example of how academic-public health system partnerships can facilitate better implementation planning at regional and national levels. For example, PP had the resources and capacity to evaluate the relative effectiveness of specific strategies that the Ministry of Health was concerned about, but for which they needed local evidence. Likewise, PP knew that sustainability was only possible if the public health system adopted and felt ownership around the intervention. Program champions and key opinion leaders were also of critical importance to the adoption and sustainment of new CCSMs: their knowledge of their context and how to work and create change within the system was key. PP obtained donations of HPV equipment and assays (GeneXpert from CEPHEID) and sample collection kits (COPAN) outside of the Ministry of Health’s budget. However, these donations helped provide key evidence of feasibility and sustained success of this CCSM approach in a challenging setting (with high cervical cancer incidence and where most transport is by boat). This has been critical to facilitating a national-level commitment to adopting this approach—with different HPV assays—throughout the country (both financially and in terms of implementation). These donations were not secured until after Phase 2 of INSPIRE, when the decision to move to HPV-based screening had already been made by stakeholders.

We have also observed important synergies between stakeholder engagement and audit/feedback strategies that are supported when using interrupted time series in LMIC contexts, as opposed to randomized designs which may distance stakeholders from a more unconstrained, real-world context, limiting the amount of actionable feedback that can be obtained. The use of qualitative research was especially useful as PP’s research team sought to understand specific factors influencing implementation, such as loss to follow-up, stigma, and acceptability of the screen-and-treat program to women [28, 32, 33, 34].

In large part, the use of ST, PAR, and IS concepts was driven by stakeholder desire to reduce health disparities, increase access to care, and ensure the sustainability of the new CCSM services, ultimately leading to a more equitable system [4]. As stakeholders mapped systemic barriers in the early phases of INSPIRE, they determined that HPV-based screening with thermal ablation follow-up at the primary level and referral to hospital only for those at greatest risk would avert the greatest number of cervical cancer cases, increasing women’s access to the health system.

The results observed in this case study have been actively disseminated through presentations, key partnerships, trainings, discussions, and engagement of key opinion leaders, as well as through passive diffusion among professional, regional, and digital networks. More data is needed regarding the mechanisms that influenced stakeholders to adopt these new CCSM practices, and how these practices may become embedded into routine practice. We are employing Normalization Process Theory [35], a sociological theory and IS framework, to explore how providers perceive the changes to the health care system and have found it useful in identifying what changes to implementation might result in improved adoption and sustainment. We are also conducting a realist evaluation to identify the underlying mechanisms that led to change [36]. We continue to measure loss-to-follow-up rates among HPV-positive women and are exploring why 30% of women—mostly women referred to the hospital—are still not making completion of care, so that we can improve on these findings [37]. Given that this is a challenge encountered by other screen-and-treat programs in Latin America, we are eager to share our own localized understanding of why this particular barrier exists and how it may be mitigated. Given the nature of this research, and the unique implementation context, this case study cannot be generalized to all cervical cancer screen-and-treat programs. However, we hope this case study will serve to provide helpful information and examples for implementers in similar contexts.

## Conclusions

Proyecto Precancer applied the INSPIRE framework to describe, guide, and analyze the implementation experience. This case study contributes to the existing body of knowledge around cervical cancer prevention by showing that task-shifting strategies to primary care were feasible and effective in this context and that the knowledge-sharing and shared decision-making enabled by the program likely resulted in higher ownership over the strategy, increasing the likelihood of positive and sustainable change. Through a renewal grant funded by the National Cancer Institute, PP is developing a multi-faceted implementation strategy and toolkit to facilitate the scale-up of context-adapted HPV-based CCSM programs in diverse contexts, including throughout Perú, and possibly Latin America.

We strongly believe that the use of IS and other interdisciplinary research approaches will generate evidence that enables rapid scale-up of effective and context-adapted CCSM programs, ultimately contributing to the global elimination of cervical cancer as a public health problem. Evidence-based interventions will offer partial solutions to help reach the WHO’s proposed elimination goals, but the most reliable progress will be achieved through the judicious and long-term use of IS strategies to drive adoption, sustainment, and scale-up of evidence-based practices within locally adapted strategies and programs.

### Supplementary Information

**Additional File 1.** IS Frameworks Employed

**Additional File 2: Table 2.** Methods, Frameworks, and Strategies by INSPIRE Phase

**Additional File 3.** SQUIRE Checklist

## Data Availability

No new data was generated or analyzed in the current case study. For information about the data described here, please contact the corresponding author.

## Abbreviations

CCSM: Cervical cancer screening and management
IS: Implementation science
MRIS: Micro Red Iquitos Sur
PP: Proyecto Precancer
TMFs: Theories, Models, and Frameworks
VIA: Visual inspection with acetic acid
