# Supplementary material for: Implementation of new technologies designed to improve cervical cancer screening and completion of care in low-resource settings: a case study from the Proyecto Precancer
Source: Implement Sci Commun. 2024 Apr 5;5:35. doi: 10.1186/s43058-024-00566-z (PMC10998344; doi:10.1186/s43058-024-00566-z)
Supplement: Supplementary file 1 — Additional File 1. IS Frameworks Employed [file 43058_2024_566_MOESM1_ESM.docx]

**Supplemental Material: Descriptions of implementation science frameworks employed**

1. ***Consolidated Framework for Implementation Research (CFIR)***

The Consolidated Framework for Implementation Research (CFIR) is an evidence-based determinant framework that describes constructs believed to influence implementation^3^. Using CFIR may allow implementers to create a better fit between interventions and the implementation context, increasing the probability of success and sustainment^3^. CFIR’s constructs fit into five broad domains, with constructs within each domain:

1. Intervention characteristics
2. The inner setting
3. The outer setting
4. Characteristics of the individuals involved in implementation
5. The process of implementation

The specific constructs and definitions for each construct can be found on CFIR’s website and in the original paper by CFIR presented in Implementation Science^3^.

Proyecto Precancer used CFIR as a sense-making and analytical device, to help surface and analyze implementation barriers and facilitators, and to select strategies for addressing these throughout the INSPIRE process. Table 2, featured in the attached supplemental file, describes how the various domains and constructs of CFIR mapped to each phase of INSPIRE, and to the implementation strategies selected as part of the INSPIRE process.

For additional reading about CFIR , the following sources may be helpful:

- Consolidated Framework for Implementation Research, *Constructs*, CFIR Research Team-Center for Clinical Management Research, 2022, cfirguide.org/constructs.
- Damschroder, Laura J et al. “Fostering implementation of health services research findings into practice: a consolidated framework for advancing implementation science.” Implementation science. 2009; 4: 50. doi:10.1186/1748-5908-4-50
- Damschroder, Laura et al. “The Consolidated Framework for Implementation Research (CFIR): progress to date, tools and resources, and plans for the future.” Implementation Science. 2015. IS vol. 10,Suppl 1 A12. doi:10.1186/1748-5908-10-S1-A12

1. ***The RE-AIM (Reach, Effectiveness, Adoption, Implementation, Maintenance) Framework***

RE-AIM is an evaluation framework that guides implementers to plan for and understand factors that may affect interventions (positively or negatively). It is one of the most widely used planning and evaluation frameworks in existence for health interventions^18^. The framework has five dimensions, which are used to guide planning and/or evaluation, improving implementation, adoption, and sustainability of programs^16^. The five dimensions are: reach, effectiveness, adoption, implementation, and maintenance.

In Proyecto Precancer’s program, RE-AIM was critical to Phase 4 of INSPIRE, contributing to iterative learning and continuous quality improvement, and indicating when program adaptation was needed (for example in data collection practices) or where evidence for sustainability was surfaced (for example, when screening continued successfully despite the setbacks of COVID).

For additional reading about the RE-AIM framework, the following sources may be helpful:

- Glasgow, R. E., Vogt Tm Fau - Boles, S. M., & Boles, S. M. (1999). Evaluating the public health impact of health promotion interventions: the RE-AIM framework. Am, J. Public Health(0090-0036 (Print)).
- Glasgow, R. E., Harden, S. M., Gaglio, B., Rabin, B., Smith, M. L., Porter, G. C., Ory, M. G., & Estabrooks, P. A. (2019). RE-AIM Planning and Evaluation Framework: Adapting to New Science and Practice With a 20-Year Review. (2296-2565 (Print)).
- RE-AIM, *What is RE-AIM?*, 2022, https://re-aim.org/learn/what-is-re-aim/
