# Supplementary material for: Implementation of new technologies designed to improve cervical cancer screening and completion of care in low-resource settings: a case study from the Proyecto Precancer
Source: Implement Sci Commun. 2024 Apr 5;5:35. doi: 10.1186/s43058-024-00566-z (PMC10998344; doi:10.1186/s43058-024-00566-z)
Supplement: Supplementary file 2 — Additional File 2: Table 2. Methods, Frameworks, and Strategies by INSPIRE Phase [file 43058_2024_566_MOESM2_ESM.docx]

Table 2. Integration of research methods, implementation science (IS) frameworks, and implementation strategies by phase of INSPIRE.

|  | INSPIRE action | Research methods utilized | IS Frameworks | Implementation strategies |
| --- | --- | --- | --- | --- |
| INSPIRE HUB | 1. Define problem situation with stakeholders 2. Launch the project | Soft systems methodology | *CFIR Domain 1*: intervention source  *CFIR Domain 5:* engaging | Build buy-in (involve existing governance structures, ID champions)  Develop relationships (build coalitions, resource-sharing agreements, formal commitments, academic partnerships) |
| Phase 1: UNDERSTAND THE SYSTEM | 1. Develop mental models of the system 2. Establish narrative and stakeholder perceptions of the system. 3. Make the system visible | AIIM  SAST stage 1 with key informant interviews and focus group discussions  KAP surveys  Audits of current system outcomes  Pathway analysis visually represented by flow charts and swim-lane diagrams | *CFIR Domain 3*: defining structural characteristics, networks, & communications  *CFIR Domain 2:* culture & implementation climate  *CFIR Domain 3:* patient needs and resources  *CFIR Domain 3:* external policies & incentives  *CFIR Domain 4:* knowledge & beliefs about the intervention  *CFIR Domain 4:* understand self-efficacy, individual stage of change and other attributes | Gather information (needs assessment, readiness to change)  Involve patient/consumers and family members  Audit current system behavior  Capture and share local knowledge |
| Phase 2: FIND LEVERAGE | 1. Engage stakeholders in group model building 2. Share, test, revise system/process maps 3. Define and localize system behaviors contributing to problem situation 4. Find leverage for change | SAST Stage 2 – design workshops  Dialectic debate and group model building (facilitated with goal to balance desirability and feasibility guided by reflection on implementation outcomes such as feasibility, cost, acceptability, sustainability, etc).  Scenario analysis | *CFIR Domain 1:* review characteristics of the intervention and options (evidence strength and quality, relative advantage, complexity, cost) & assess adaptability and trialability of alternatives  *CFIR Domain 3:* assess cosmopolitanism, peer pressure, influence of external policies/incentives  *CFIR Domain 4:* assess KAB about intervention options  *CFIR Domain 4:* Group level stage of change | Assess readiness and identify barriers  Get feedback from audit of current system behavior  Purposefully re-examine the intervention  Tailor strategies to overcome barriers and honor preferences  Model and simulate change  Conduct local consensus discussions  Distribute educational materials and conduct educational meetings  Make training/education dynamic and participatory  Inform local opinion leaders  Create a learning collaborative  Consider restructuring strategies as leverage opportunities  Consider financing strategies as leverage opportunities  Mandate change |
| Phase 3: ACT STRATEGICALLY | 1. Stakeholder designed implementation plan 2. Infrastructure modifications, training, dissemination plan development 3. Implement changes | Work group soft systems methodology with research team facilitation | *CFIR Domain 1:* design quality  *CFIR Domain 1:* complexity  *CFIR Domain 5:* planning  *CFIR Domain 5:* executing | Develop a formal implementation blueprint  Tailor strategies to overcome barriers and honor preferences  Stage implementation scale-up  Involve patients/consumers and family members  Recruit, designate, and train for leadership  Obtain formal commitments  Develop effective educational materials relevant to mandated change  Develop a glossary of implementation (including new models)  Distribute educational materials  Conduct ongoing, dynamic training  Conduct educational outreach visits  Use train-the-trainer strategies  Provide ongoing consultation  Place new interventions on fee for service lists/formularies  *Develop supply chain management*  Revise professional roles  Create new clinical teams  Change services sites  Change equipment  Change records systems  Develop and organize quality monitoring systems  Develop tools for quality monitoring  Use advisory boards and work groups  Conduct cyclical tests of change  Create or change credentialing and/or licensure standards |
| Phase 4: LEARN AND ADAPT | 1. Ongoing M&E using stakeholder defined implementation outcome metrics 2. Share M&E with stakeholder group 3. Re-initiate INSPIRE cycle where indicated by identification and localization of new or unresolved problem situation | M&E for primary implementation outcomes  SAST with KII and FGD  Design workshops | RE-AIM  CFIR Domain 5: Reflecting and evaluating | Provide ongoing consultation  Sustain a learning collaborative  Use mass media to increase reach *(only after system behavior is stabilized post-implementation)*  Use advisory boards and working groups  Organize clinical implementation team meetings |

Reproduced from *Cancer Epidemiology, Biomarkers & Prevention.* 2020. Vol.29,9, 1710-1719, Gravitt, P. et al, “Integrative Systems Praxis for Implementation Research (INSPIRE): An Implementation Methodology to Facilitate the Global Elimination of Cervical Cancer., with permission from the American Association for Cancer Research (AACR).
